# Detecting Freezing of Gait in Parkinson Disease Using Multiple Wearable Sensors Sets During Various Walking Tasks Relative to Medication Conditions (DetectFoG): Protocol for a Prospective Cohort Study

**DOI:** 10.2196/58612

**Published:** 2025-02-06

**Authors:** Sébastien Cordillet, Sophie Drapier, Frédérique Leh, Audeline Dumont, Florian Bidet, Isabelle Bonan, Karim Jamal

**Affiliations:** 1 Physical and Rehabilitation Medicine Department Clinical Investigation Center INSERM 1414 University Hospital of Rennes Rennes France; 2 Neurology Department Clinical Investigation Center INSERM 1414 University Hospital of Rennes Rennes France

**Keywords:** Parkinson, detection, freezing of gait, sensor, wearable, freezing, walk, neurodegenerative, movement

## Abstract

**Background:**

Freezing of gait (FoG) is one of the most disabling symptoms of Parkinson disease (PD). Detecting and monitoring episodes of FoG are important in the medical follow-up of patients to assess disease progression and functional impact and to adjust treatment accordingly. Although several questionnaires exist, they lack objectivity. Using wearable sensors such as inertial measurement units (IMUs) to detect FoG episodes offers greater objectivity and accuracy. There is no consensus on the number and location of IMU, type of algorithm, and method of triggering and scoring the FoG episodes.

**Objective:**

The objective of this study is to investigate the use of multiple wearable sensors sets to detect FoG in patients with PD during various walking tasks under different medication conditions.

**Methods:**

This single-center, prospective cohort study (DetectFoG) will include 18 patients with PD. Patients will be fitted with 7 IMUs and will walk a freezing-provoking path under different tasks—“single task,” “dual motor task,” or “dual cognitive task”—and medical conditions corresponding to levodopa medication (“on” or “off”). Passages will be videotaped, and 2 movement disorder specialists will identify FoG episodes in the videos. The accuracy, sensitivity, specificity, positive predictive value, and negative predictive value of the most effective combination of wearable sensors for detecting FoG episodes will be studied.

**Results:**

The study is currently in the data collection phase, having commenced recruitment in February 2024. Once all data have been gathered, the data analysis will commence. As of August 2024, 3 patients have been recruited. It is anticipated that the results will be published by the end of 2025.

**Conclusions:**

Detecting FoG episodes in various medical and clinical settings would provide a more comprehensive understanding of this phenomenon. Furthermore, it would enable reliable and objective monitoring of the progression of this symptom based on treatments and the natural course of the disease. This could serve as an objective tool for monitoring patients and assessing the severity and frequency of FoG.

**Trial Registration:**

Clinicaltrials.gov NCT05822258; https://www.clinicaltrials.gov/study/NCT05822258

**International Registered Report Identifier (IRRID):**

DERR1-10.2196/58612

## Introduction

Affecting more than 8.5 million people worldwide in 2019, Parkinson disease (PD) is the second most common neurodegenerative disease [1]. PD is diagnosed using criteria from the UK Parkinson’s Disease Society Brain Bank [2] and is defined by the cardinal symptoms of tremor, bradykinesia, rigidity, and postural instability, along with other motor and nonmotor symptoms [3].

Among the various motor symptoms, freezing of gait (FoG), defined as a “brief and episodic absence or marked reduction in the forward progression of the feet despite the intention to walk” [4], is one of the most disabling symptoms of PD. In advanced and severe forms of PD, FoG occurs in 50% to 80% of cases [5]. FoG is correlated with PD severity and disease duration [6]. It increases the risk of falls [7] and loss of independence and affects patients’ quality of life [8].

The detection of FoG episodes is an important issue for patient follow-up and treatment adjustment. Various subjective and objective methods can be used to assess these symptoms. Daily completion of a motor diary by the patient is one possibility for assessing these symptoms, but it relies on the patient’s subjective judgment and is often discontinued after a few days or is not feasible in the presence of cognitive impairment [9]. Objective scores with predefined exercises (eg, double spot walking, 180° turning, etc) are available to assess FoG episodes during medical consultations; however, these exercises do not always trigger FoG episodes due to several reasons. First, the phenomenon known as the “white coat effect,” whereby patients may perform differently in the presence of medical professionals, can alter their natural response and reduce the likelihood of FoG episodes [10]. Second, the structured nature of clinical assessments often causes a switch from automatic to goal-directed pathways, reducing the occurrence of FoG episodes, which typically occurs in more automatic walking scenarios [11]. Finally, the predefined exercises are constrained by time, unfamiliarity, and the artificiality of the clinical environment, which can influence patients’ natural gait patterns. Thus, they fail to provide accurate information on the frequency and severity of FoG episodes in daily life [11]. One potential alternative method involves videotaping patients in different contexts and conducting a postscored examination, considered the gold standard, although this approach demands time and expertise [11].

To overcome these limitations, wearable sensors, such as electromyographs, electroencephalography electrodes, or inertial measurement units (IMUs), provide a solution for automatic FoG detection. The combination of accelerometer and gyroscopes data from an IMU seems to be the most widely used solution with high performance (sensitivity: 86%; specificity: 92.9% [12]). This compact sensor is easy to install and allows FoG assessment both in clinical practice and during daily living [9,13].

Despite numerous studies and literature reviews on the use of these wearable sensors for FoG detection, there is as yet no consensus in the literature on the optimal methodology for their use. It is likely that this discrepancy stems from the inherent variability in the protocols used. Indeed, there is considerable heterogeneity in the protocols for triggering [11] (medical condition: “on” or “off” levodopa treatment; freezing-provoking path; and dual-task conditions). In fact, there are few studies that directly compare FoG episodes in “on” and “off” conditions with small cohorts and a low number of participants [11,12,14]. Furthermore, many locations including feet, shin or ankle, thigh, and pelvis have been investigated [15]. The use of multiple sensors provides a more detailed and holistic view of the patient’s movements, enabling the capture of subtle changes that may not be detectable with a single sensor [15]. An IMU is cost-effective and straightforward to wear, rendering it suitable for both laboratory evaluation and daily use. Even if patients prefer to wear sensors solely at the wrist, such as a stopwatch, or conceal them beneath clothing at the ankle or on a belt (lower back), as demonstrated by O’Day et al [15], the placement of sensors on multiple body parts could enhance the robustness of FoG detection across various walking tasks and different FoG subtypes. Furthermore, examining FoG under dual-task conditions provides a more comprehensive understanding of how cognitive load impacts freezing episodes, which only a few studies have addressed [16-20]. Finally, several other variables may confound the study, including the wide age range of cohorts (aged 7-118 years) and the diverse range of FoG episodes (50-1110) [21-23]. Therefore, this study is innovative as it stands out for its ability to replicate findings across various conditions, particularly the medical condition. Furthermore, it explores the effects of dual-task scenarios and optimal sensor placement in a larger cohort than those found in the literature, significantly enhancing our understanding of the underlying mechanisms. These multiple dimensions provide a more nuanced and robust perspective, allowing for better generalization of the results and improving practical applications in medical settings.

Regardless of the wearable sensors used or the freezing protocol, the accuracy of detection is sensitive to the type of algorithm [9,11,12,23]. Yet, the algorithms used to detect FoG episodes are controversial, especially for real-time detection. The threshold method provides a straightforward approach to the implementation and interpretation of algorithms. In this context, the freezing index—defined as the ratio between the power bands of freezing (3-8 Hz) and locomotion (0.5-3 Hz)—is the most commonly used feature in threshold-based algorithms [9,11,12,23]. This single feature accurately detects many FoG episodes (sensitivity: 84.3%; specificity: 78.4% [24]), but detection fails when no motion is observed and voluntary stops may be mistakenly classified as FoG [17]. Adding multiple indices can enhance algorithm performance, but it also increases complexity and the difficulty of tuning thresholds [25,26]. To address these limitations, machine learning (ML) algorithms have been developed to improve FoG detection performance. Indeed, ML algorithms could better fit the model to increase the accuracy of the models; this is a subfield of artificial intelligence that gives an algorithm the ability to learn without being explicitly programmed [12]. Among the various ML algorithms, support vector machine, multilayer perceptron, and ensemble classifiers (eg, random forest or AdaBoost) have proven to be the most efficient. Determining the best ML algorithm is challenging due to the highly heterogeneous nature of the training data. A limitation of ML algorithms is that training the model and processing the data can require high computational cost. Fortunately, advances in computer technology now offer the possibility of early or real-time detection with ML models [12].

The primary objective is to assess the accuracy of the optimal combination of wearable sensors for detecting FoG episodes in patients with PD. The secondary objectives of this study include measuring the specificity, sensitivity, positive predictive value (PPV), and negative predictive value (NPV) of the optimal combination of wearable sensors for detecting FoG episodes. Regarding the setup, we also aim to compare its performance (accuracy, sensitivity, specificity, PPV, and NPV) across various clinical and medical conditions.

## Methods

### Study Design and Participants

This study is a single-center, prospective cohort trial (DetectFoG) involving patients with PD with FoG at the University Hospital of Rennes. Patients will be recruited from the neurology department of University Hospital of Rennes during their visit to the neurologist. This study aims to include a total of 18 patients. To mitigate potential issues such as withdrawal of consent, absence of FoG during neurologist annotation, loss of follow-up (failure to attend the second visit), or inability to complete the required number of passages (fewer than 6), initially, 20 patients will be enrolled. Participants experiencing any of these issues will be excluded from the study.

Inclusion criteria will include patients older than 18 years diagnosed with PD according to the UK Brain Bank criteria. Patients must self-report as freezers, scoring between 1 and 3 on question 13 of the Movement Disorder Society-Unified Parkinson’s Disease Rating Scale (MDS-UPDRS) II [27], and be capable of walking 30 m without assistance to complete the freezing-provoking path. Exclusion criteria will apply to patients scoring less than 20 out of 30 on the Montreal Cognitive Assessment [28] who are unable to give informed consent and those with neurological, orthopedic, or rheumatic comorbidities that could impact gait and ability.

### Ethical Considerations

The study design was approved by the ethical committee of Ile de France III (23.01067.000298-MS01) on October 1, 2023. All data collection will adhere to applicable guidelines and regulations. Participants will provide informed consent before enrolling in the study.

### Protocol

**Figure 1:**
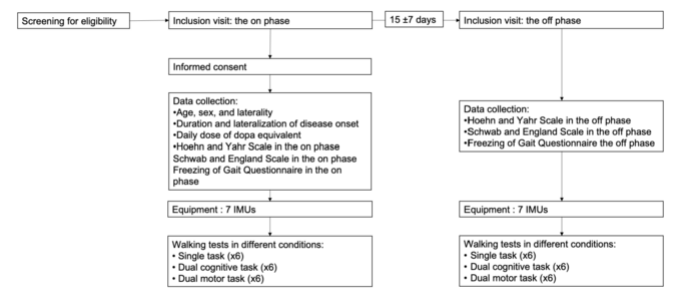
Timeline of study. IMU: inertial measurement unit.

After verifying the inclusion and exclusion criteria, the neurologist will notify the principal investigator (KJ). The principal investigator will then contact the patients to provide comprehensive and understandable information about the study’s objectives, as well as their right to refuse participation or withdraw at any time. If the patients consent and sign the consent form, they will participate in 2 visits separated by 2 weeks (±7 days), encompassing both “on” and “off” phases of levodopa treatment (Figure 1).

For organizational purposes, the first visit (inclusion visit) will occur during the “on” phase of levodopa medication, while the second visit (follow-up visit) will be during the “off” phase. During the “on” phase, patients will be assessed when oral treatment maximally improves dopa-sensitive PD symptoms (best “on” state, typically 1-2 hours after ingestion). This state will be determined based on patients’ subjective assessment, similar to their daily motor self-assessments (home diaries), which has been established as reliable in various studies [29-31]. Assessment during the “off” phase will occur after 12 hours without treatment, preferably in the morning before the first levodopa dose.

**Figure 2:**
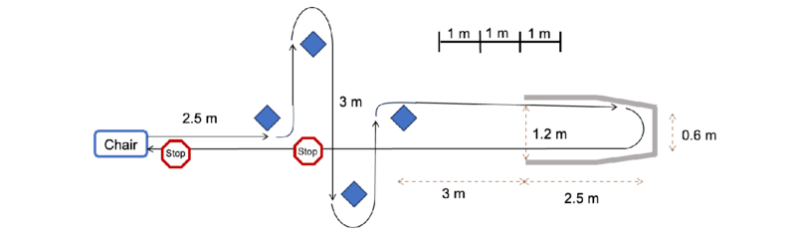
Freezing-inducing path (adapted from Pardoel et al [32], which is published under Creative Commons Attribution 4.0 International License [33]).

During each visit, participants will walk on a freezing-provoking path at a comfortable speed under 3 different clinical conditions (single task [ST], dual cognitive task [DCT], and dual motor task [DMT]). The Pardoel freezing-provoking path, known to induce FoG episodes [32], includes standing up, walking with a slalom (left and right turns), navigating a narrow passage with a 180° turn, and returning straight, with 2 designated stops—one chosen by the patient and another in front of a chair (Figure 2).

Participants will undergo testing under different dual-task conditions, which are recognized for promoting FoG episodes [32]. The DMT involves walking with a ball on a tray within a drawn circle, keeping the ball centered. The DCT requires participants to generate as many words as possible starting with a specified letter. Each participant will complete a maximum of 6 blocks, with each block consisting of the 3 conditions (ST, DCT, and DMT), resulting in a total of 18 trials (6 blocks × 3 conditions per block). The sequence of conditions within each block will be randomized to mitigate any order effects. This protocol accommodates varying endurance levels among patients; some may complete each block quickly with few or no FoG episodes, while others may take longer due to frequent tremors that consume energy. This flexible approach ensures that the study is comprehensive and respects each patient’s physical limitations. However, participants who complete fewer than 2 blocks (6 trials) will be excluded from the analysis to ensure adequate data collection.

### Data Collection (and Preprocessing)

#### Clinical Data

Various patient data are collected, including age, sex, laterality of symptoms, duration of PD, lateralization of symptom onset, daily dose of levodopa equivalent, MDS-UPDRS III score [27], Freezing of Gait Questionnaire (FoGQ; “on” and “off” phases) [34], Hoehn and Yahr Scale (“on” and “off” phases) [35], and Schwab and England Scale (“on” and “off” phases) [36]. The FoGQ is used to assess the characteristics of FoG. The Hoehn and Yahr Scale and the Schwab and England Scale serve as tools for the overall evaluation of patients with PD: the former to gauge disease severity and the latter to evaluate daily life impact, functional status, and level of dependence.

#### FoG Measurement

##### IMU Measurements

**Figure 3:**
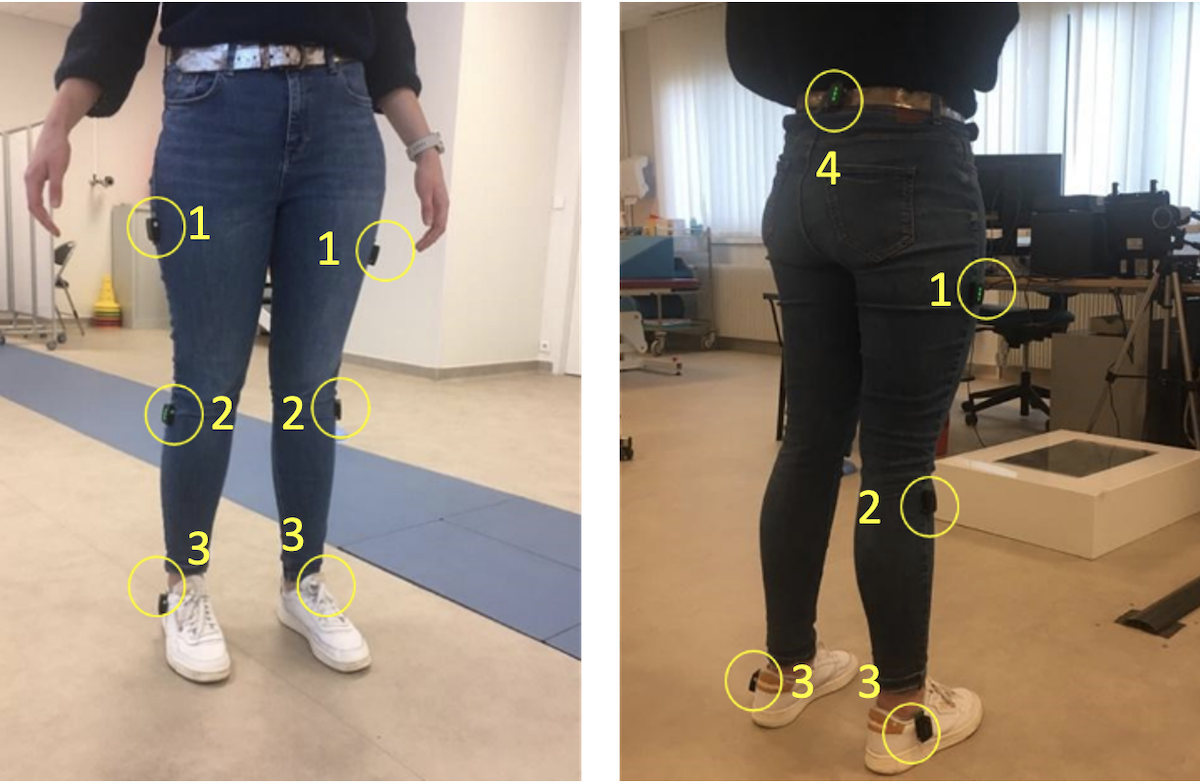
Inertial measurement unit placements. 1: Thighs (side, upper third); 2: fibulas (upper third); 3: foot (below lateral malleolus); 4: Lumbar (L5 level).

Patients will wear the Trigno Avanci IMU (Delsys; Figure 3), which includes a triaxial accelerometer, gyroscope, and magnetometer. Data will be transmitted via Bluetooth and collected using the Delsys application programing interface and then transferred to QTM 2020 software (Qualisys). The software manages recording at a sampling frequency of 148.15 Hz and synchronizes with cameras. While this sampling frequency is higher than that in some studies, it has been previously used, similar to the approach taken by Camps et al [37]. This frequency is selected to ensure that all nuances associated with FoG, particularly micromovements, are captured. IMU data will also undergo windowing with a 2-second window length and a 0.4-second shift. A trained operator will position the sensors manually, aligning them with anatomical axes. Seven IMUs will be placed as follows: 1 on each thigh (lateral side, upper third), 1 on each fibula (upper third), 1 on each foot (below the lateral malleolus), and 1 at lumbar level (L5).

##### Video Recording and Window Labeling

During each visit, and during trials designed to induce freezing episodes, patients will be recorded using 14 synchronized Miqus M3 cameras (Qualisys). These cameras will record at a resolution of 1824 × 1088 pixels with a frequency of 25 Hz. FoG episodes will be identified from the videos by 2 movement disorders specialists (FL and SD) independently using QTM 2020 software (Qualisys). The start of a freezing episode is defined as “the moment when the stepping foot does not leave the ground despite a clear intention to step,” and the end is defined as “the moment when the stepping foot begins or resumes an effective step” [32]. In cases where there is disagreement about the presence of a FoG episode, the specialists will discuss and reach a consensus. Following the methodology of Pardoel et al [32], the data timeline will be segmented into 2-second windows with a 0.4-second shift between windows (80% overlap). Windows will be labeled as “FoG window” when the entire window corresponds to a period of FoG. Windows not meeting this criterion, either during periods without FoG or during transitions between FoG episodes and non-FoG periods, will be labeled as “no-FoG window.” The relative duration of FoG will be assessed by calculating the ratio of FoG windows to the total number of windows. This ratio provides a measure of the freezing time while accounting for the window labeling method used. To ensure comparability with other studies and to mitigate the effect of varying labeling methods across research [32,38], we will also calculate the total number of FoG episodes and the cumulative freezing time based on onset and end events, categorized by task type, session, and patient. This approach will facilitate meaningful comparisons with existing literature on FoG detection methodologies.

### Data Management

All data are securely stored in a dedicated folder on a local server with restricted access limited to the research team. Patient’s clinical information (such as age, sex, laterality of symptoms, duration of PD, lateralization of symptom onset, daily dose of levodopa equivalent, FoGQ score, Hoehn and Yahr Scale rating, Schwab and England Scale assessment, and MDS-UPDRS III score) along with study results (videos and IMU data) are collected and controlled by the University Hospital of Rennes. A database containing video data is duplicated for each expert responsible for labeling FoG episodes. Time annotations will be exported as events in C3D files along with IMU data before the windowing process, contributing to a final database used for subsequent data analysis. This structured approach ensures the integrity and confidentiality of patient data while facilitating precise event annotation and thorough analysis of collected data.

### Data Analysis

#### Features Extractions

Each window of data will undergo normalization to achieve zero mean and unit variance. Our study distinguishes itself through an exhaustive exploration of features aimed at identifying FoG episodes. From each window, signal features will be extracted from both the time and frequency domains. A comprehensive array of features will be included, such as the number, duration, and length of acceleration or angular velocity reversals, kurtosis, and skewness [14]. Furthermore, we will incorporate metrics such as the magnitude of mean and SD of signals, as well as specific metrics related to smoothness such as spectral arc length measure [39] and the freezing ratio [40]. Nonlinear modeling features, such as sample entropy or Lyapunov exponent, which quantify gait regularity and complexity, will also be used [41]. Furthermore, measures of inter- and intralimb coordination will be integrated to highlight specific limb dynamics during FoG episodes. Feature selection will be conducted using Relief-F, chosen for its suitability in our application over methods such as minimum redundancy maximum relevance ranking [20]. This approach ensures that the selected features are highly relevant to distinguishing FoG episodes, thereby optimizing the accuracy and effectiveness of our analysis.

#### Model Training

**Figure 4:**
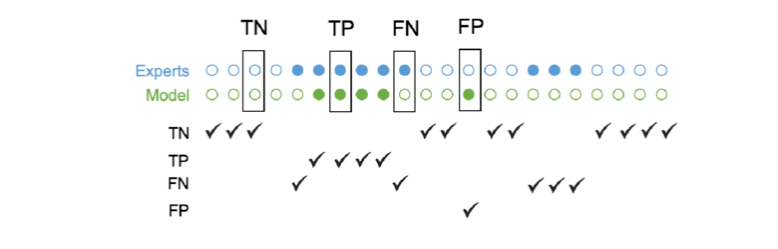
Segmentation on true negatives (TNs), true positives (TPs), false negatives (FNs), and false positives (FPs) for each second according to the experts and the model.

The top-ranked features identified through Relief-F will be used in a decision tree (DT) model for classifying windows of data. The DT model will be tested using 5 and 10 decision splits and with various sets of top-ranked features ranging from 5 to 20. The performance of the DT model will be evaluated using metrics such as accuracy, sensitivity, specificity, PPV, and NPV. The model’s performance will be determined based on the classification of each window as true positive (TP), true negative (TN), false positive (FP), or false negative (FN), comparing the model’s classifications with the experts’ identification of FoG episodes on video recordings (Figure 4).

The agreement between the model and experts regarding FoG or non-FoG periods will be assessed as follows:

- TP: A FoG period correctly identified by both the experts and the model
- TN: A non-FoG period correctly identified by both the experts and the model
- FP: A FoG period incorrectly identified by the model but not by the experts
- FN: A FoG period incorrectly identified by the experts but not by the model

Performance scores will be calculated using the following equations:



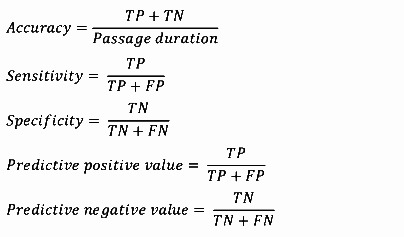



#### Model Validation

The model validation will use the leave-one-subject-out (LOSO) cross-validation technique to assess its performance across participants and accommodate the considerable variability between participants. While some studies [20,42] advocate for the leave-one-freezer-out (LOFO) cross-validation technique to avoid overrepresentation of patients without FoG in training and validation sets, our study focuses on participants who have experienced at least 1 episode of FoG during their initial visit. Consequently, each patient will have FoG episodes, making the LOSO approach more suitable for our validation process.

### Statistical Analysis

Clinical data of the participants will be presented as means (SD) or medians with IQRs for continuous data, depending on their distribution. Categorical data will be reported as counts (with percentages within parentheses).

#### Primary Outcome

The main objective of our study is to identify the most relevant features and locations of wearable sensors for detecting FoG episodes. The Relief-F technique will rank features extracted from data collected by all sensor segments. The top-ranked features across all subjects will indicate the most critical sensors for detecting FoG episodes, and these features will be used in model training and validation. The LOSO method will be used for validation, and accuracy will be computed for each subject. Furthermore, Relief-F analysis will be conducted individually for each subject to potentially reveal redundancy in the top-ranked features across subjects. Feature extraction, selection, model training, and validation will be implemented using Python 3.0 (PythonLabs) scripts.

#### Secondary Outcomes

We will investigate the differences in model performance across various conditions to propose recommendations for interpreting the model. Accuracy, sensitivity, and specificity will be evaluated using a mixed-effects model with fixed effects for patients and random effects for the type of condition (ST, DCT, and DMT) and medication state (“on” and “off”).

## Results

The study is currently in the data collection phase, with recruitment starting in February 2024. Once all data have been gathered, data analysis will commence. As of August 2024, 3 patients have been recruited. We anticipate publishing the results by the end of 2025. The results of this study will be presented at scientific events and published in scientific journals.

## Discussion

### Strengths and Limitations of This Study

The aim of this study is to assess the accuracy of the most effective combination of wearable sensors in detecting FoG in patients with PD. This setup has been developed following an extensive literature review and clinical evaluation of patients.

To date, consensus on sensor placement remains elusive, necessitating a detailed examination of individual and collective placements [15]. The model used will incorporate a broad array of features from both temporal and frequency domains documented in the literature to maximize accuracy. Moreover, this model will undergo testing on a sizable cohort of patients (n=18), each completing 18 passages on a designated freezing-inducing path, yielding a comprehensive data set. Furthermore, the model will be evaluated on a path known to provoke FoG under different medical (“on” and “off” levodopa medication) and clinical (ST, DMT, and DCT) conditions [32]. This evaluation aims to simulate various walking tasks akin to daily life and assess their potential to induce or alleviate FoG, particularly in dual-task scenarios. Several datasets are available, some with restricted accessibility. Multimedia Appendix 1 outlines several pertinent and comparable datasets. Our dataset’s strength lies in its incorporation of multiple sensor placements across varied medication states and task complexities, including cognitive dual tasks. This dataset will be openly accessible or supplied on request, fostering research and collaboration in the field.

One limitation of this study is its reliance on a controlled environment. Despite efforts to simulate ecological tasks that mimic everyday challenges and tend to provoke gait freezes (FoG) under dual-task conditions, it is crucial to acknowledge that the study is conducted under controlled conditions. For future research, conducting tests in real-life settings over prolonged periods would be advantageous. This approach could facilitate the accumulation of larger datasets across diverse situations, thereby enhancing our understanding of FoG in more complex real-world scenarios.

### Perspectives

The miniaturization of sensors is unlocking new opportunities for monitoring patients with PD, particularly in detecting episodes of FoG. Extending this capability across all medical and clinical settings would offer a more comprehensive understanding of this phenomenon, which remains incompletely understood. Moreover, it would facilitate dependable and objective monitoring of symptom progression, influenced by treatments and the disease’s natural trajectory. This could serve as an objective tool for patient monitoring, assessing both the severity and frequency of FoG.

Objective evaluation is crucial for identifying suitable medical and nonmedical treatments, including rehabilitation or deep brain stimulation [43]. Research has underscored the significance of personalized treatment tailored to individual patient needs and circumstances [44]. Real-time detection facilitates the integration of FoG episode identification with external stimuli, such as sensory, auditory, or visual cues, offering a significant opportunity to mitigate FoG episodes [45].

### Conclusions

This study aims to enhance the detection of FoG in patients with PD through the use of multiple wearable sensors. By evaluating various sensor placements and the accuracy of detection algorithms, the study aims to determine the most effective combination of wearable sensors for detecting FoG episodes. Despite the study being conducted in a controlled laboratory environment rather than real-life settings, the findings are expected to significantly advance understanding and monitoring of FoG. The anticipated results will provide crucial insights into optimal sensor configurations and detection methodologies, ultimately supporting the development of more precise and personalized treatment strategies for patients with PD. Publication of these results by the end of 2025 will contribute valuable data to ongoing efforts aimed at improving patient care and enhancing quality of life for individuals affected by PD.

## Appendix

Multimedia Appendix 1 Comparative overview of datasets for freezing-of-gait evaluation.

## Abbreviations

DCT: dual cognitive task
DMT: dual motor task
DT: decision tree
FN: false negative
FoG: freezing of gait
FoGQ: Freezing of Gait Questionnaire
FP: false positive
IMU: inertial measurement unit
LOFO: leave-one-freezer-out
LOSO: leave-one-subject-out
MDS-UPDRS: Movement Disorder Society-Unified Parkinson’s Disease Rating Scale
ML: machine learning
NPV: negative predictive value
PD: Parkinson disease
PPV: positive predictive value
ST: single task
TF: true negative
TN: true positive

## Data Availability

No data are currently available, but the results of the trial will be disseminated either with all the accompanying dataset or on request from the authors.
