# Supplementary material for: Detecting Freezing of Gait in Parkinson Disease Using Multiple Wearable Sensors Sets During Various Walking Tasks Relative to Medication Conditions (DetectFoG): Protocol for a Prospective Cohort Study
Source: JMIR Res Protoc. 2025 Feb 6;14:e58612. doi: 10.2196/58612 (PMC11843059; doi:10.2196/58612)
Supplement: Multimedia Appendix 1 [file resprot_v14i1e58612_app1.docx]

| **Author** | **DOI** | **Patients (% with freezing** | **provoking FOG protocol** | **Sensors** | **placement** | **Various walking tasks contain turn** | **Various walking tasks containts narrow space** | **Compare ON/OFF** | **Compare double task** | **open access** |
| --- | --- | --- | --- | --- | --- | --- | --- | --- | --- | --- |
| Bachlin et al 2010 | 10.1109/TITB.2009.2036165 | 10 (80%) | Lab | acc,gyro | lowerback  knee  ankle | yes | yes | no | no | yes |
| Zhang2022 | 10.1038/s41597-022-01713-8 | 12(100%) | Lab | acc,gyro | lowerback  ankles  wrists | yes | yes | no | no | yes |
| Ribeiro et al 2023 | 10.3389/fnins.2022.832463 | 35(100%) | Lab | acc,gyro | shank | yes | no | no | yes | yes |
| Yang2024 | 10.1186/s12984-024-01320-1 | 12(92%) | Lab | acc,gyro | lowerback  shank  foot | yes | yes | yes | yes | no |
| Cockx et al 2023 | 10.1186/s12984-023-01175-y | 14(100%) | Lab | acc | ankle  knee | yes | yes | no | no | yes |
| O'Day et al. 2022 | 10.1186/s12984-022-00992-x | 7(100%) | Lab | acc,gyro | lowerback  shank  foot  chest | yes | yes | NS | NS | yes |
| O'Day et al. 2022 | 10.1186/s12984-022-00992-x | 4(100%) | Lab | acc,gyro | lowerback  thigh  shank  foot  chest  wrist | yes | yes | NS | NS | yes |
| Salomon et al. 2024 | 10.1038/s41467-024-49027-0 | 57(100%) | Lab | acc,gyro | lowerback | yes | yes | yes | yes | yes |
| Salomon et al. 2024 | 10.1038/s41467-024-49027-0 | 45(100%) | Home | acc,gyro | lowerback | yes | yes | yes | yes | yes |
| Salomon et al. 2024 | 10.1038/s41467-024-49027-0 | 65(69%) | Daily | acc,gyro | lowerback | NA | NA | NA | NA | yes |
| Reches et al. 2020 | 10.3390/s20164474 | 71(100%) | Lab | acc,gyro | lowerback  ankles | yes | yes | yes | yes | no |
| Rodríguez-Martín et al. 2017 | 10.1371/journal.pone.0171764 | 21(NS) | Home | acc,gyro | waist | yes | yes | yes | no | no |
|  |  |  |  |  |  |  |  |  |  |  |
| **Our dataset** |  | **20(100%)** | **Lab** | **acc,gyro** | **lowerback**  **thigh**  **shank**  **foot** | **yes** | **yes** | **yes** | **yes** | **yes** |

NA: not application , NS : not specified

1.

Bächlin M, Plotnik M, Roggen D, Maidan I, Hausdorff JM, Giladi N, et al. Wearable assistant for Parkinson’s disease patients with the freezing of gait symptom. IEEE Trans Inf Technol Biomed. 2010 Mar;14(2):436–46.

2.

Cockx H, Nonnekes J, Bloem BR, van Wezel R, Cameron I, Wang Y. Dealing with the heterogeneous presentations of freezing of gait: how reliable are the freezing index and heart rate for freezing detection? Journal of NeuroEngineering and Rehabilitation. 2023 Apr 27;20(1):53.

3.

O’Day J, Lee M, Seagers K, Hoffman S, Jih-Schiff A, Kidziński Ł, et al. Assessing inertial measurement unit locations for freezing of gait detection and patient preference. Journal of NeuroEngineering and Rehabilitation. 2022 Feb 13;19(1):20.

4.

Reches T, Dagan M, Herman T, Gazit E, Gouskova NA, Giladi N, et al. Using Wearable Sensors and Machine Learning to Automatically Detect Freezing of Gait during a FOG-Provoking Test. Sensors. 2020 Jan;20(16):4474.

5.

1.

Ribeiro De Souza C, Miao R, Ávila De Oliveira J, Cristina De Lima-Pardini A, Fragoso De Campos D, Silva-Batista C, et al. A Public Data Set of Videos, Inertial Measurement Unit, and Clinical Scales of Freezing of Gait in Individuals With Parkinson’s Disease During a Turning-In-Place Task. Front Neurosci. 2022 Feb 23;16:832463.

6.

Rodríguez-Martín D, Samà A, Pérez-López C, Català A, Arostegui JMM, Cabestany J, et al. Home detection of freezing of gait using support vector machines through a single waist-worn triaxial accelerometer. PLOS ONE. 2017 Feb 15;12(2):e0171764.

7.

Salomon A, Gazit E, Ginis P, Urazalinov B, Takoi H, Yamaguchi T, et al. A machine learning contest enhances automated freezing of gait detection and reveals time-of-day effects. Nat Commun. 2024 Jun 6;15(1):4853.

8.

Yang PK, Filtjens B, Ginis P, Goris M, Nieuwboer A, Gilat M, et al. Freezing of gait assessment with inertial measurement units and deep learning: effect of tasks, medication states, and stops. J NeuroEngineering Rehabil. 2024 Feb 13;21(1):24.

9.

Zhang W, Yang Z, Li H, Huang D, Wang L, Wei Y, et al. Multimodal Data for the Detection of Freezing of Gait in Parkinson’s Disease. Sci Data. 2022 Oct 7;9(1):606.
